# A *meta*-analysis of the long-term efficacy of Amantadine for Levodopa-induced dyskinesia in Parkinson’s disease

**DOI:** 10.1016/j.prdoa.2025.100405

**Published:** 2025-11-01

**Authors:** Kayla Williams, Maurice A. Curtis, Lisa Gombinsky, Priya Parmar

**Affiliations:** a Department of Statistics, the University of Auckland, Auckland, New Zealand; b Department of Anatomy and Medical Imaging, and Centre for Brain Research, the University of Auckland, Auckland, New Zealand; c Unruly Company, Neuro and Rehab Specialized Personal Training and Conductive Education, New Zealand

**Keywords:** Parkinson’s disease, Drug therapy, Amantadine, Levodopa-induced dyskinesia, Neurological manifestations, Systematic review, Meta-analysis

## Abstract

**Introduction:**

This systematic review and *meta*-analysis investigated the long-term efficacy of Amantadine for Levodopa-induced dyskinesia (LID) in Parkinson’s disease patients. Our analysis focused on longer-term motor outcomes, including those which are under-reported in the literature.

**Methods:**

We identified relevant articles by searching four online databases and reviewing citations. The primary outcomes included the Unified Parkinson’s Disease Rating Scale (UPDRS) or Movement Disorder Society Unified Parkinson’s Disease Rating Scale (MDS-UPDRS) Part IV and daily “ON” time; secondary outcomes included UPDRS/MDS-UPDRS III and IVa, Unified Dyskinesia Rating Scale (UDysRS) total score, and daily “OFF” time. For each outcome, we conducted *meta*-analyses for numerous time-points after beginning treatment: “up to 13 weeks”, “up to 16 weeks”, “12 to 16 weeks”, and “38 to 101 weeks”. Our outcome measure was the Standardized Mean Differences (SMDs) between the pooled Amantadine-Levodopa and Levodopa only (control) groups.

**Results:**

We found associations between Amantadine and each motor outcome. The most considerable was an SMD of −0.73 points for Amantadine compared to control for the UPDRS/MDS-UPDRS IV at up to 16 weeks after beginning treatment (*P* < 0.0001). However, the magnitude of most associations decreased with time, indicating declining long-term efficacy. We also identified a risk of added “adverseness”; 11 studies had patient-reported adverse events, mainly overlaid with known symptoms.

**Conclusion:**

Our findings suggest that Amantadine is an effective adjunct medication for improving LID and broader Parkinson’s disease symptoms. However, evidence of its declining long-term efficacy and “adverseness” have important implications for its use in Parkinson’s disease therapy.

## Highlights

• Amantadine linked to reduced Levodopa-induced dyskinesia in Parkinson’s disease.
• Improvement in numerous motor outcomes in Parkinson’s disease patients.
• Prolonged treatment may result in reduced efficacy of Amantadine in the long term.
• Risk of adverse events with Amantadine therapy, beyond expected symptoms.

## 1 Introduction

Parkinson’s disease (PD) is a neurological and movement disorder associated with motor and non-motor disability [1]. The disease causes dopamine-producing neurons in the brain’s substantia nigra to deteriorate, resulting in motor deficiencies such as rest tremor, stiffness, and bradykinesia, or slowed movement [2]. Common non-motor symptoms include constipation, chronic pain, an increased risk of depression and anxiety, and declining cognition and memory [1].

Levodopa has been a clinical leader for PD treatment since its development as a dopamine therapy in the mid-1960s [3]. Levodopa naturally occurs in the brain as a dopamine precursor. The oral medication provides the remaining functioning dopaminergic neurons with the dopamine precursor, which is then converted to dopamine. This helps to address the dopamine deficiency which contributes to PD symptoms [4]. However, Levodopa’s end-of-dose and peak effects cause fluctuation in dopamine levels, leading to motor fluctuation and unwanted side effects [4].

Such a side effect is dyskinesia, which is defined as erratic and involuntary spasmic movements. Primary dyskinesia becomes more probable as PD progresses because the dopamine receptors and/or dopaminergic cells can no longer effectively uptake the required dopamine [5]. However, when its onset or worsening is attributed to prolonged Levodopa treatment, it is considered Levodopa-induced dyskinesia (LID) [6]. As Levodopa is taken multiple times in a day, there is a combination of fluctuating dopamine levels and gradual degradation of dopaminergic neurons within the nigrostriatal pathway in the brain’s basal ganglia. Over time, this contributes to LID [7].

Patients fluctuate between the “ON” state, where mobility and function are relatively good, and the “OFF” state, which is characterized by immobility and worsening of parkinsonian symptoms [8]. While the severity and duration of the “ON” and “OFF” cycle differs by individual, PD progression often leads to more rapid fluctuations with worsening symptoms [8]. A poor understanding of LID pathophysiology has resulted in limited clinical options for its treatment and management [9].

Amantadine is another effective medication used in PD treatment. As a dopamine agonist for the *N*-methyl-*D*-aspartate (NMDA) receptor, it regulates activity in the brain’s subthalamic nucleus [10]. Amantadine mimics dopamine through both enhancing dopamine release from nerve terminals and delaying its re-uptake. Current literature suggests that alongside this, the medication also suppresses the re-uptake of serotonin and adrenaline, while promoting the release of noradrenaline. This is associated with increased peripheral sympathetic activity [11]. It is often used to support dopamine therapy (Levodopa), which itself replaces dopamine rather than mimicking it [4]. Amantadine also blocks NMDA activity in the striatum, which may reduce excitotoxicity and therefore be slightly protective of striatal neurons that respond to dopamine input [12].

Previous literature suggests that when Amantadine is taken adjunct to Levodopa, it is effective in reducing LID in PD patients [13]. However, the evidence is somewhat limited. Our literature search yielded only three relevant large-scale *meta*-analyses, all of which were published prior to 2018 and included a relatively small selection of trials. The most recent, Kong [13], was published in 2017, and at least five new randomized controlled trials (RCTs) have been published since. Those previous *meta*-analyses focused primarily on PD rating scale outcomes, such as the Unified Parkinson’s Disease Rating Scale (UPDRS), and analysed data from a maximum of 13 weeks’ treatment. We designed a *meta*-analysis that would address this by focusing on longer-term Amantadine efficacy (longer than 13 weeks) and include under-reported motor outcomes. These include the daily number of hours people with PD spend in the “ON” and “OFF” states. As outcomes, these provide a different perspective on efficacy and are often more meaningful to those with PD compared to rating scales. Our aim was to provide clinically meaningful findings for both clinicians and those with PD, contributing to a more comprehensive understanding of Amantadine as a clinical intervention for LID.

## 2 Methods

### 2.1 Literature search strategy

**Fig. 1:**
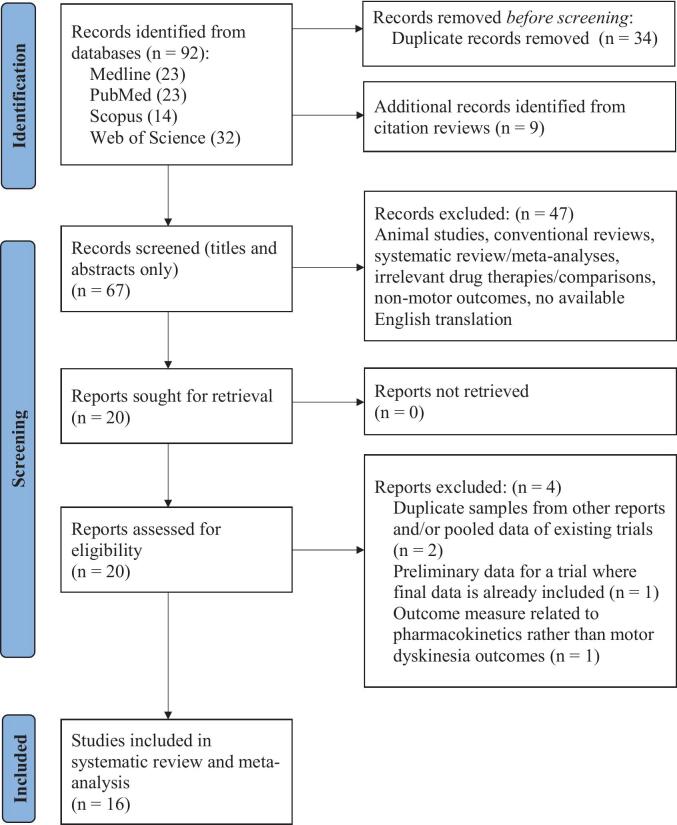
PRISMA flow chart depicting the literature selection and screening process.

We conducted a literature search across Medline, PubMed, Scopus, and Web of Science databases for randomized controlled trials (RCTs) investigating the efficacy of Amantadine in treating LID (Fig. 1). Search terms included: Amantadine* AND (“Parkinson’s disease” OR pd) AND (Levodopa* OR l-dopa*) AND (“random* control* trial” OR “random* clinical trial”). We restricted the publication period to be between 12/31/2004 and 12/31/2024 but did not restrict language, participant characteristics, document type, or trial length. Relevant studies were also identified by reviewing article citations and previous *meta*-analyses.

### 2.2 Study selection criteria

The inclusion criteria for our *meta*-analysis were as follows: an RCT study design involving PD patients, an exposure of Amantadine adjunct (with Levodopa) compared to Levodopa alone, and a motor outcome for LID. We excluded papers that conducted animal experiments, were published in languages other than English, had a *meta*-analysis, review, or editorial format, or had an irrelevant exposure or outcome.

### 2.3 Data extraction

We extracted the following information from each selected article: first author, publication year, study design, trial name (if applicable), stage of PD targeted, Amantadine formulation and dosage, study length and follow-up periods, number of participants, characteristics of participants (including age, gender, and years since PD diagnosis), motor outcomes of interest, adverse events, primary and secondary objectives of the trial, mean or mean difference from control, and a measure of variability, typically standard deviation (SD), standard error (SE) or 95 % CI.

**Fig. 2:**
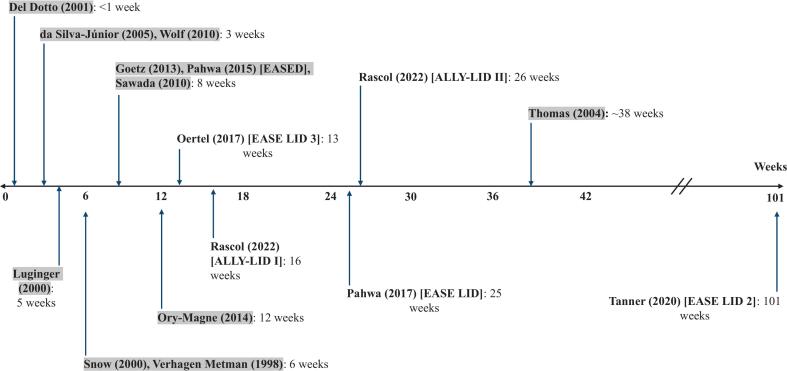
Timeline showing durations of trials selected to be in the *meta*-analysis. The horizontal axis represents trial duration in weeks, and each vertical arrow represents one or more studies at a given time-point. Study authors, trial names, and durations are included at the end of the corresponding arrow. Highlighted study names represent those also included in Kong (2017).

For studies involving Amantadine at multiple dosages, we included only the arm with the dosage that was most comparable to that of other trials or part of the primary outcome. To facilitate analysis and address variation in follow-up, we grouped data into time-points based on clinically important periods (Fig. 2). When studies collected and reported data from multiple periods of the trial, we allocated each value to the closest time-point. Available data and the Cochrane Handbook for Systematic Reviews were used to calculate standardized mean differences (SMDs) when the required means and SDs were not directly reported.

### 2.4 Data analysis

We did not have access to the original trial data, and used reported summaries of means, SDs, sample sizes, and SMDs in our *meta*-analysis. SMDs were used for all continuous outcomes with 95 % CIs and *P*-values to investigate Amantadine efficacy. Because trials reported the same motor outcome but used different methods, the use of SMDs standardized the results to ensure they are comparable and to produce a pooled estimate. Heterogeneity of the studies was investigated using a χ2 test and I^2^ statistic along with the relevant *P*-value. Based on heterogeneity and sample sizes of the included studies, we used a random effects model with the inverse variance method for each continuous outcome. Statistical significance was assessed using two-sided *P*-values where the level of statistical significance was *P* < 0.05. We used forest plots to visualize the association between Amantadine use and the outcome of interest. The Shapiro-Wilk test was used to test normality of data (minimum n = 252) in each *meta*-analysis (Supplementary Table S2).

For the outcome with the most included studies, namely UPDRS/MDS-UPDRS IV at up to 16 weeks, we performed sub-group analyses (Supplementary Fig. S3). This investigated whether dosage, trial design, or Amantadine formulation contributed to heterogeneity or otherwise impacted our results.

In addition, we conducted sensitivity analyses for the same outcome to assess the robustness and quality of the evidence. This included sub-grouping the studies by their quality based on the RoB 2 tool (Supplementary Fig. S5.a) and a “leave-one-out” analysis (Supplementary Fig. S6). Finally, we generated funnel plots to assess publication bias (Supplementary Fig. S10). All analyses were conducted using R version 2024.12.0 + 467 [14].

## 3 Results

### 3.1 Study characteristics

101 records were initially identified in the literature search. After removal of duplicates and title and/or abstract screening, 20 studies satisfied the inclusion criteria and were deemed relevant. Of these, two were excluded because they provided pooled data from studies we had already selected [15,16]. One paper was excluded due to providing interim data for the same trial as another more recent paper, and a fourth was excluded as its outcome measures were irrelevant.

We selected 16 studies for *meta*-analysis. Each was published between 1998 and 2022, and their durations range from less than one week to 101 weeks (Fig. 2). The newer studies, not included in Kong [13], were longer in duration and had potential to provide insight into longer-term Amantadine efficacy.

Of these studies, five were a randomized cross-over design [[17], [18], [19], [20], [21]]. The remaining 11 were a randomized parallel-group design [[22], [23], [24], [25], [26], [27], [28], [29], [30], [31]]. While Rascol [27] is one publication, it reports data for both ALLY-LID I and II, which are identical in methodology but unique in participant pool and time frame. They have therefore been analysed as two separate studies in the present study.

The pooled sample includes 837 participants, all of whom are PD patients experiencing LID. 363 participants underwent Amantadine treatment, 377 received control, and 97 received both in cross-over studies. The sample consists of 383 males, 298 females, and 156 individuals of other or unknown gender. The mean age of participants was 63.7 years (range 59 to 68 years), and the mean number of years since PD diagnosis was 10.7 (range 8.4 to 17.8 years). The selected studies were relatively consistent in dosages, ranging from 100 mg/day at the beginning of the trials to 200–420 mg/day in later stages (Supplementary Fig. S1). Various Amantadine formulations, including ADS-5102, Amantadine hydrochloride, Amantadine chloridrate, OS320 immediate release/extended release, Amantadine sulfate, and intravenous injection, were included (Supplementary Fig. 1).

Nine of the 16 studies reported the UPDRS or the updated Movement Disorder Society Unified Parkinson’s Disease Rating Scale (MDS-UPDRS), which focuses on motor function such as facial recognition, gait, rigidity, and speech. Eleven reported Part IV, which focuses on motor complications such as dyskinesia and dystonia. Seven reported the Unified Dyskinesia Rating Scale (UDysRS), which measures the severity, frequency, and impact of dyskinesias on daily life for PD patients. Daily “ON” time (hours) with and without troublesome dyskinesias were reported by five and four studies respectively, while daily “OFF” time (hours) was reported by seven. Along with the commonly reported UPDRS/MDS-UPDRS and UDysRS outcomes, we also included daily “ON” and “OFF” time, which are relatively underreported. Data for each outcome were allocated into the time-points “up to 13 weeks”, “up to 16 weeks”, “12 to 16 weeks” and “38 to 101 weeks”. This facilitated comparison of Amantadine efficacy in the short- and longer-term.

### 3.2 Primary efficacy outcomes

**Fig. 3:**
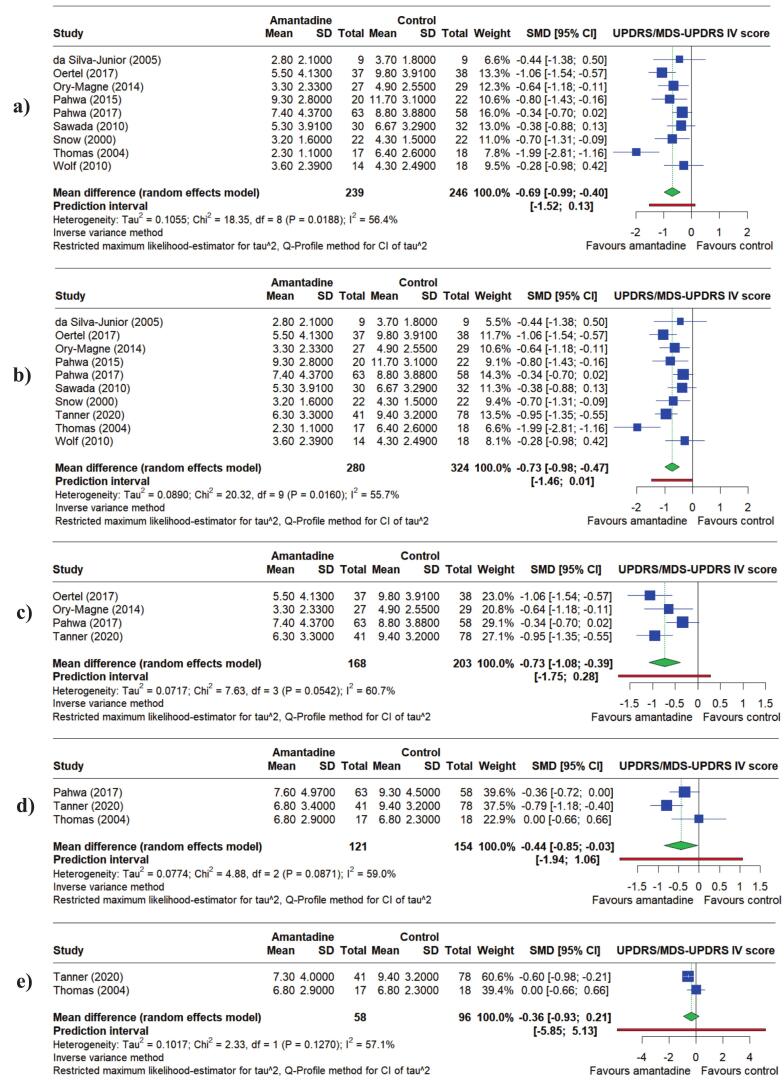
Forest plots showing the Standardized Mean Differences (SMDs) for the effect of Amantadine compared to placebo on the UPDRS/MDS-UPDRS IV score for: a) up to 13 weeks, b) up to 16 weeks, c) 12 to 16 weeks, d) 24 to 38 weeks and e) 38 to 101 weeks.

The UPDRS/MDS-UPDRS is used clinically to diagnose PD, evaluate progression, and determine treatment. In the present study, Amantadine had a beneficial effect on UPDRS/MDS-UPDRS IV mean score. The Standard Mean Differences (SMDs) for Amantadine compared to control were: −0.69 points (95 % Confidence Interval (95 % CI) = −0.99 to −0.40, *P* < 0.0001) up to 13 weeks, −0.73 points up to 16 weeks (95 % CI = −0.98 to −0.47, *P* < 0.0001), −0.73 points at 12 to 16 weeks (95 % CI = −1.08 to −0.39, *P* < 0.0001), −0.44 points at 24 to 38 weeks (95 % CI = −0.85 to −0.03, *P* = 0.0372) and −0.36 points at 38 to 101 weeks (95 % CI = −0.93 to 0.21, *P* = 0.2149) (Fig. 3). These SMDs indicate a negative association between Amantadine use and mean Part IV score, meaning that Amantadine may reduce dyskinesia and improve movement in patients. However, this effect decreased in both magnitude and significance over time.

**Fig. 4:**
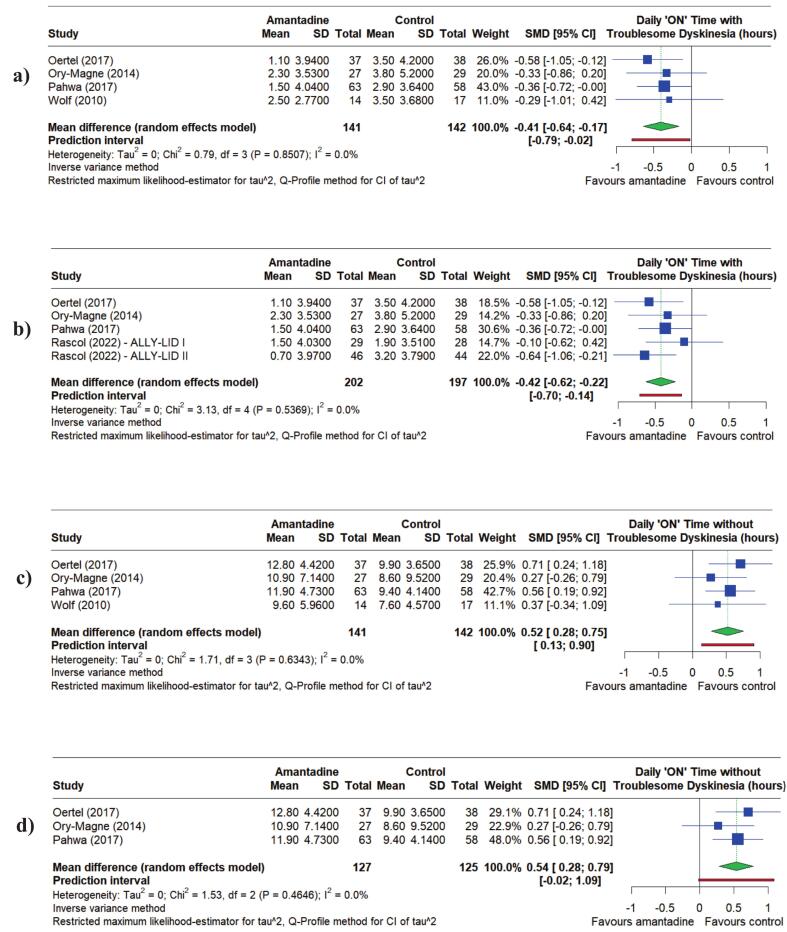
Forest plots showing the Standardized Mean Differences (SMDs) for the effect of Amantadine compared to placebo on: a) Daily “ON” time with troublesome dyskinesia (hours) up to 13 weeks, b) Daily “ON” time with troublesome dyskinesia (hours) at 12 to 16 weeks, c) Daily “ON” time without troublesome dyskinesia (hours) up to 13 weeks, and d) Daily “ON” time without troublesome dyskinesia (hours) at 12 to 16 weeks.

Daily “ON” time in hours also provides insight into Amantadine efficacy. In the literature, the minimal clinically important difference for “ON” time with and without troublesome dyskinesia are 0.5 and 1 h respectively [32]. In the present study, those treated with Amantadine experienced a reduced mean daily “ON” time with troublesome dyskinesia (in hours) compared to control. At up to 13 weeks, the SMD for the daily “ON” time with troublesome dyskinesia for Amantadine compared to control was −0.41 h (95 % CI = −0.64 to −0.17, *P* = 0.0008), and at 12 to 16 weeks this was −0.42 h (95 % CI = −0.62 to −0.22, *P* < 0.0001) (Fig. 4). Conversely, the SMDs for daily “ON” time without troublesome dyskinesia were 0.52 h at up to 13 weeks (95 % CI = 0.28 to 0.75, *P* < 0.0001) and 0.54 h at 12 to 16 weeks (95 % CI = 0.28 to 0.79, *P* < 0.0001) (Fig. 4). This suggests that Amantadine reduced patients’ time in the “ON” state with substantial dyskinesia each day, translating to more time with optimal mobility, motor control, and independence. There was no substantial change in the magnitude of either effect over time.

### 3.3 Secondary efficacy outcomes

Secondary outcomes included UPDRS/MDS-UPDRS Part III, Part IVa, and the UDysRS, which are clinically relevant but secondary due to data limitations. Amantadine was negatively associated with each outcome. For UPDRS/MDS-UPDRS Part IVa (dyskinesias only), the SMDs for the mean score for Amantadine compared to control were: −0.78 points at up to 13 weeks (95 % CI = −1.10 to −0.46, *P* < 0.0001) and −0.72 points at 12 to 16 weeks (95 % CI = −0.98 to −0.47, *P* < 0.0001). The former SMD was −0.9 points higher than Part IV at the same time-point, but the effects were similar at 12 to 16 weeks. For Part III, the SMD for the mean score was −0.27 points at up to 13 weeks (95 % CI = −0.51 to −0.03, *P* = 0.027). The Shapiro-Wilk test indicated deviation from normality (*P* = 0.01 for control), and available data was limited, so further analyses were not conducted. For UDysRS, the SMDs for the mean score were: −0.69 points at up to 13 weeks (95 % CI = −1.10 to −0.27, *P* = 0.0011), −0.58 points at up to 16 weeks (95 % CI = −0.89 to −0.28, *P* = 0.0002), and −0.48 points at 12 to 16 weeks (95 % CI = −0.77 to −0.19, *P* = 0.0013). Amantadine reduced the mean score for each scale, which is linked to reduced dyskinesia and improved motor function for patients. However, both UPDRS/MDS-UPDRS IVa and UDysRS showed moderately decreased SMDs over time, indicating that this effect decreased in magnitude over time.

The daily “OFF” time in hours was also analysed. While it is less relevant to dyskinesia than “ON” time, it can still provide evidence of Amantadine efficacy. The SMDs for mean daily “OFF” time for Amantadine compared to control were −0.35 h up to 13 weeks (95 % CI = −0.65 to −0.06, *P* = 0.0181), −0.30 h up to 16 weeks (95 % CI = −0.49 to −0.11, *P* = 0.0016), and −0.19 h at 12 to 16 weeks (95 % CI = −0.39 to 0, *P* < 0.0001). There is evidence of a negative association between Amantadine and daily “OFF” time. This suggests it may decrease the time patients spend in the “OFF” state each day, increasing their time being mobile and independent. However, this effect decreases in magnitude over time. The SMD at 12 to 16 weeks is noticeably smaller than up to 16 weeks, suggesting that the shorter studies included in the latter may contribute to a larger effect size.

### 3.4 Adverse events

Safety and adverse events were also a secondary outcome of our analysis. Of the 16 studies reported, 11 included patient-reported adverse events; however, many of these were also known PD symptoms. Headaches, confusion, changes in mood and mental status, and worsening dyskinesia were reported as AEs by four of the 16 studies. Urinary retention and urinary tract infections were reported by five studies. Six of the studies reported sleep disturbances, excessive sweating, skin reactions including livedo reticularis, nausea and/or vomiting, oedema, and dizziness as AEs. Furthermore, constipation, dry mouth, hallucinations and/or psychosis, and imbalance and/or falls were reported by seven, eight, nine, and ten of the analysed studies, respectively. The overlap in reported AEs across trials shows how Amantadine treatment has the risk of creating added “adverseness” for PD patients beyond their expected PD symptoms (Supplementary Table S1).

### 3.5 Quality assessment and sensitivity analyses

The Cochrane Handbook for Systematic Reviews and its Risk of Bias 2 (RoB 2) tool were used to assess study quality and risk of bias. The 16 studies were established to be of medium-to-high quality. Three studies had a low risk of bias in the five RoB 2 domains. Eight were of “some concern” due to minor issues in at least one domain. Most commonly, this was due to missing data or bias in which outcomes were reported or made available. The remaining three studies had a high overall risk of bias. We allocated this rating due to a high risk of missing data in reported outcomes [20], a high risk of bias through lack of randomization and allocation blinding [28], or minor bias issues in three domains (selection bias of reported outcomes, missing data, and randomization) [19]. The risk of bias for specific studies is discussed in the Supplementary Fig. S10 legend.

Numerous sub-group and sensitivity analyses were performed to assess the robustness and quality of the evidence. Detailed output for these analyses can be found in the Supplementary Materials. We selected the single analysis with the most available data to act as a representative for all analyses. This was UPDRS/MDS-UPDRS IV at up to 16 weeks, which included ten out of the 16 studies (Fig. 3). Firstly, we performed sub-group analyses by dosage (“high” and “low”, with 200 mg/day as the threshold) and trial design (cross-over or parallel). Neither test for sub-group differences were statistically significant (*P* = 0.2571 and *P* = 0.2780 respectively), meaning these factors are unlikely to affect the associations (Supplementary Fig. S3). However, there was statistically significant evidence for a difference between Amantadine formulations (ranging from 0.28 for Amantadine sulfate to 1.99 for Amantadine chloridrate [singular studies] with *meta*-analyses of ADS-5102 (0.76) vs. Amantadine hydrochloride (0.50) *P =* 0.01071 – Supplementary Fig. S4). Secondly, we performed sensitivity analysis based on study quality. Studies were grouped into “low quality” (a rating of “some concern” for two or more RoB 2 domains, or a rating of “high” risk for one) and “high quality” (a “some concern” rating for one domain, or all “low” risk). There was again no statistically significant difference between the quality groups differences (*P* = 0.4689 – Supplementary Fig. S5). In addition, we performed a “leave-one-out” sensitivity analysis by removing studies one at a time and observing the impact on the association with UPDRS/MDS-UPDRS IV mean score. The negative association was unchanged and all SMDs were similar (*P* < 0.0001 for each – Supplementary Fig. S6). Finally, visual inspection of funnel plots showed no substantial asymmetry to suggest publication bias (Supplementary Fig. S10). While Thomas (2004) was observed to have larger SMDs than the other studies, it was included for consistency with Kong [12]. From these results, we conclude that no study or factor, other than Amantadine formulation, had an undue influence on our analyses.

## 4 Discussion

This *meta*-analysis provides evidence that Amantadine is effective in reducing LID with a negative association between Amantadine treatment and UPDRS/MDS-UPDRS and UDysRS mean scores. Because these scales measure the degree to which PD and dyskinesia impact the individual, a lower score indicates reduced LID in patients. A negative SMD, which was found in all outcomes, shows that the pooled Amantadine group received a lower mean score than that of the pooled control group. This was particularly strong for the UPDRS/MDS-UPDRS Part IV mean score, a primary outcome. The results show a 0.5 to 1 point change in rating scales (Supplementary Table S1). This can reflect a dramatic change for PD patients, as a 1-point difference may represent a symptom worsening from mild to moderate or moderate to severe. In this way, these point differences quantify changes in patients’ ability to perform daily activities and function independently.

Similarly, our analysis found that Amantadine was negatively associated with both daily “OFF” time and daily “ON” time with dyskinesia, and conversely, positively associated with “ON” time without troublesome dyskinesia. Therefore, Amantadine is associated with reduced time spent in the “OFF” state and reduced time with substantial dyskinesia in the “ON” state. While the reported changes in “ON” and “OFF” time were between 0 and 1 h daily, this translates to up to an hour of increased mobility and motor function each day for patients (Supplementary Table S1). This, in turn, supports their ability to perform daily tasks and move independently for longer periods.

However, the beneficial effect of Amantadine reduced in magnitude for most outcomes as time increased. This is shown through decreasing SMDs for almost every successive time point. A smaller SMD suggests that the two groups’ mean values are closer together, so a trend of decreasing SMDs indicates that the values for the Amantadine and control groups become more similar over time. While the final UPDRS/MDS-UPDRS IV SMD at 38 to 101 weeks is not statistically significant, it still provides an indication of a decreasing association. This suggests that Amantadine’s efficacy in treating LID may wane over time. We also note that for the secondary outcome “OFF” time, the SMD at 12 to 16 weeks is considerably smaller than that at up to 16 weeks. The latter analysis includes shorter studies, which are more likely to report large Amantadine effects than studies in analyses with longer follow-up (i.e. less than 12 weeks vs.12 to 16 weeks).

Our findings have immediate implications for clinical use. The dyskinesia outcomes are unique in being both clinician- and patient-oriented. Improvements in the UPDRS/MDS-UPDRS and UDysRS are informative for a physician, while changes in “ON” and “OFF” times (previously under-reported) are particularly meaningful to a patient. These results have been summarized and reported in Supplementary Table S1. They have the potential to support clinicians, neurologists, PD patients and their caregivers in making informed decisions about PD treatment.

The limitations of this study include a lack of long-duration data, significant heterogeneity among the included studies, and a lack of individual clinical trial data. These arise from the nature of the data available to us. We found very little data beyond 25 weeks of Amantadine treatment for LID, and existing studies reported few outcomes beyond rating scales and “ON” or “OFF” time − further evidencing the need to assess Amantadine’s longer-term efficacy. This corresponds to dosages needing to increase over time, which in turn increases the number and severity of adverse events and side effects. Data on adverse events can not be formally integrated into statistical tests, and a more in- depth assessment of clinical impact and quality of life would be required. We detail here, however, a descriptive summary of the reported risk of added adverseness with Amantadine from the known literature (Supplementary Table S1). Given no data is available across trials to analyze the impact of these adverse events, we recommend new studies focus on formal reporting of adverse events and research meaningful ways to minimize adverse events and side effects that come with increases in Amantadine dosage.

As we found evidence for a statistically significant difference in motor outcomes between Amantadine formulations, this should also be a focus. Finally, future studies should incorporate more individual clinical trial data once these become available.

The evidence collected here consolidates what is known about Amantadine efficacy but also presents important long-term efficacy considerations. It provides opportunities for future research on alternative therapies and more effective ways to administer Amantadine, whilst considering confounding effects such as comorbidities (and medications) that become more prevalent with age. It also justifies investigation into patients who are female, younger (<65 years), older (>75 years), and identifying as different ethnic groups. Early onset PD impacts 30 % of patients, meaning they live with PD symptoms for longer and are at higher risk of medications becoming ineffective earlier. Exploration of the “reboot effect”, where patients undergo a wash-out period and re-start medication, could address these concerns and illustrate Amantadine’s anti-dyskinetic and long-duration effects [24]. We recommend future studies record measurements of quality of life, mental health, sleep, and fatigue, as well as motor parameters, for a more clinically relevant view and analysis.

Parkinson’s disease is the second most common neurological disease worldwide and has the fastest-growing rates of prevalence and disability. These will continue to increase with global population ageing; projections estimate PD will impact 25.2 million people by 2050 [33]. Therefore, the development of effective and safe long-term dopamine and adjunct therapies is vital. An improved understanding of non-motor outcomes, such as patient quality-of-life, mental health, sleep, and fatigue, along with adverse events, will contribute to more effective clinical intervention for LID and a broad range of PD symptoms.

## CRediT authorship contribution statement

**Kayla Williams:** Writing – review & editing, Writing – original draft, Visualization, Software, Investigation, Formal analysis, Data curation. **Maurice A. Curtis:** Writing – review & editing, Supervision, Project administration, Methodology, Funding acquisition, Conceptualization. **Lisa Gombinsky:** Writing – review & editing, Visualization. **Priya Parmar:** Writing – review & editing, Visualization, Validation, Supervision, Resources, Project administration, Methodology, Investigation, Funding acquisition, Data curation, Conceptualization.

## Declaration of competing interest

The authors declare that they have no known competing financial interests or personal relationships that could have appeared to influence the work reported in this paper.

## Data Availability

The datasets generated and/or analysed during the current study, and underlying code, are available in the GitHub repository “Williams_Amantadine_LID_Meta_Analysis”, and can be accessed via this link: https://github.com/kayla-williams/Williams_Amantadine_LID_Meta_Analysis.
