# Supplementary material for: A meta-analysis of the long-term efficacy of Amantadine for Levodopa-induced dyskinesia in Parkinson’s disease
Source: Clin Park Relat Disord. 2025 Nov 1;13:100405. doi: 10.1016/j.prdoa.2025.100405 (PMC12639294; doi:10.1016/j.prdoa.2025.100405)
Supplement: Supplementary Data 1 [file mmc1.docx]

**Supplementary Materials**

**Study Dosage Timeline**

**
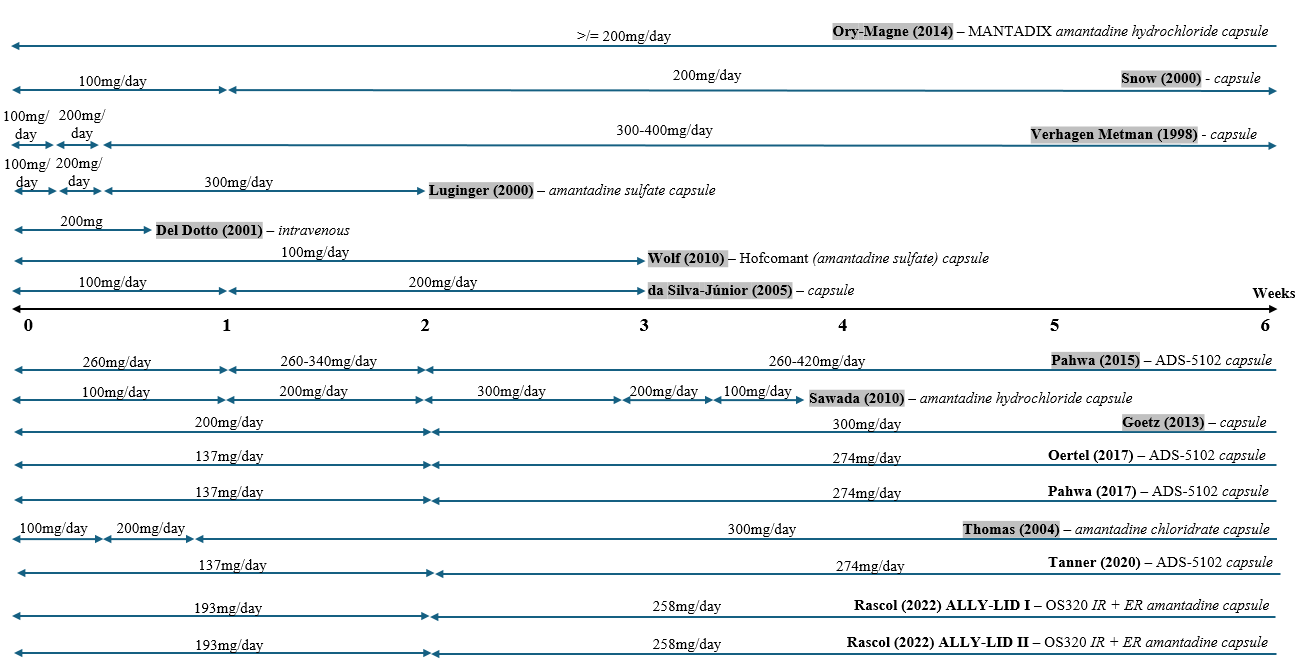
**

**Fig. S1. *Timeline*** ***showing dosages of trials selected to be in the meta-analysis and change over time****. The horizontal axis represents study duration in weeks, and each study is shown horizontally with blue arrows. Each arrow is denoted with the dosage administered in the trial during the respective time period. For cross-over and/or wash-out studies, only the length of the active treatment period for any given patient is illustrated in this Figure. Trials shaded in grey represent those also included in Kong et al. (2017).*

**Meta-Analysis Forest Plots**

*UPDRS/MDS-UPDRS III*


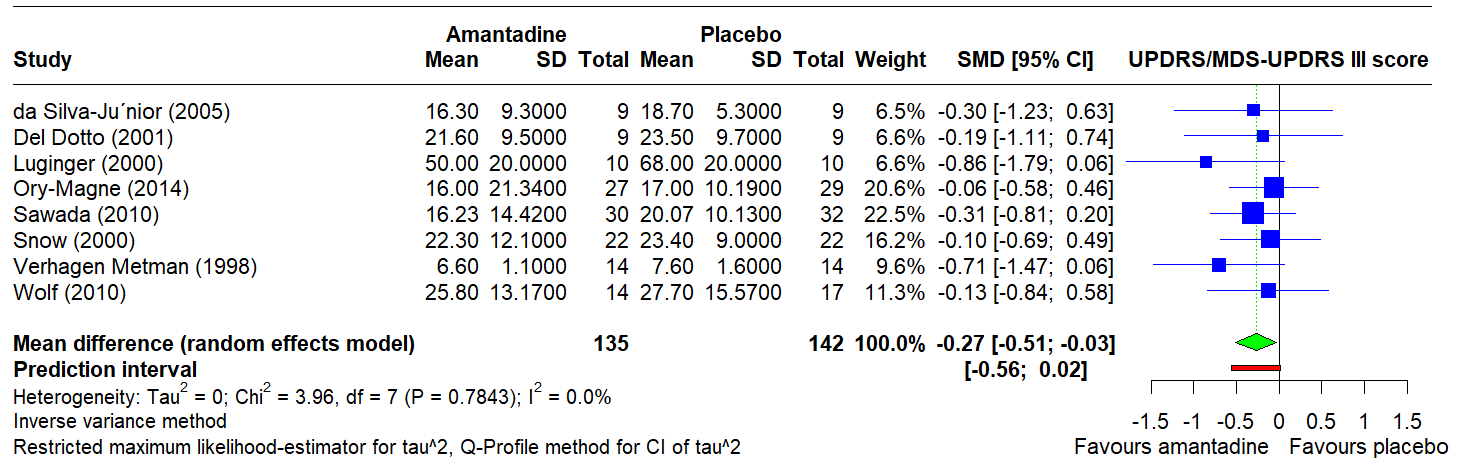

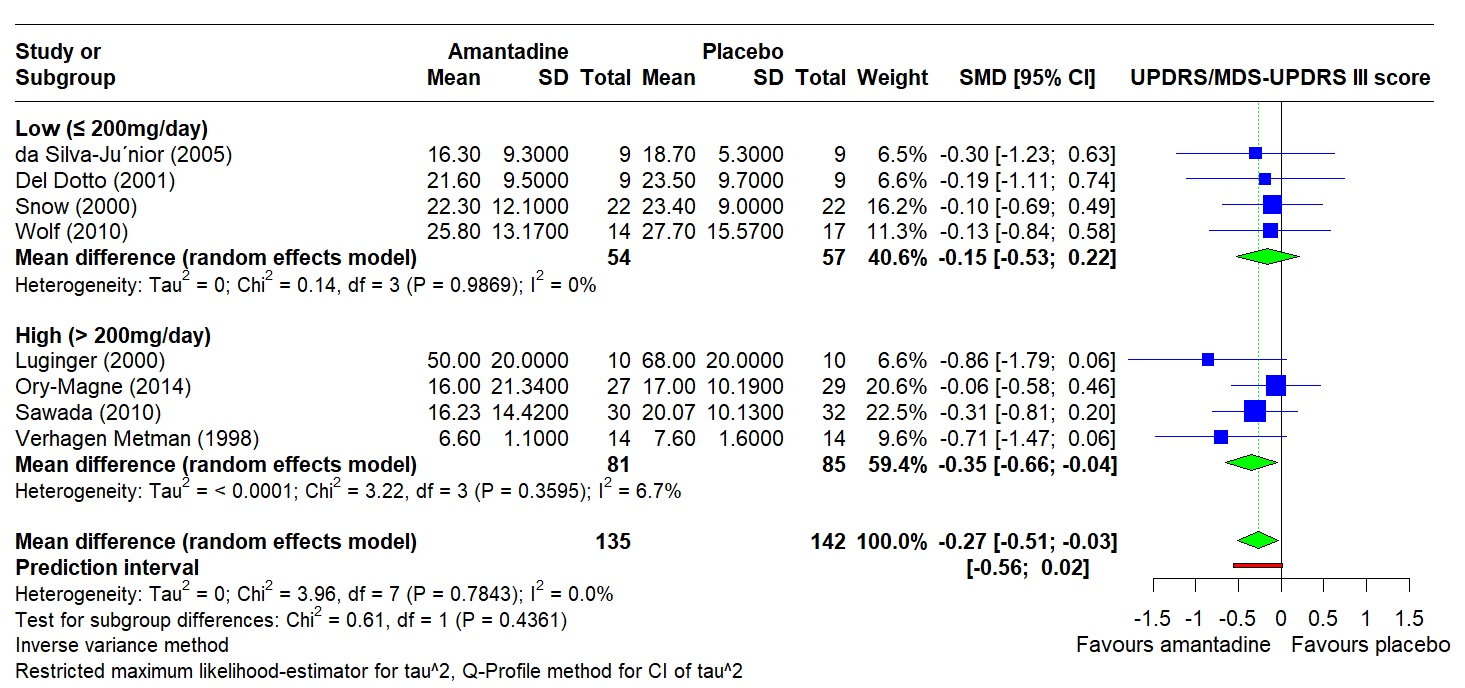

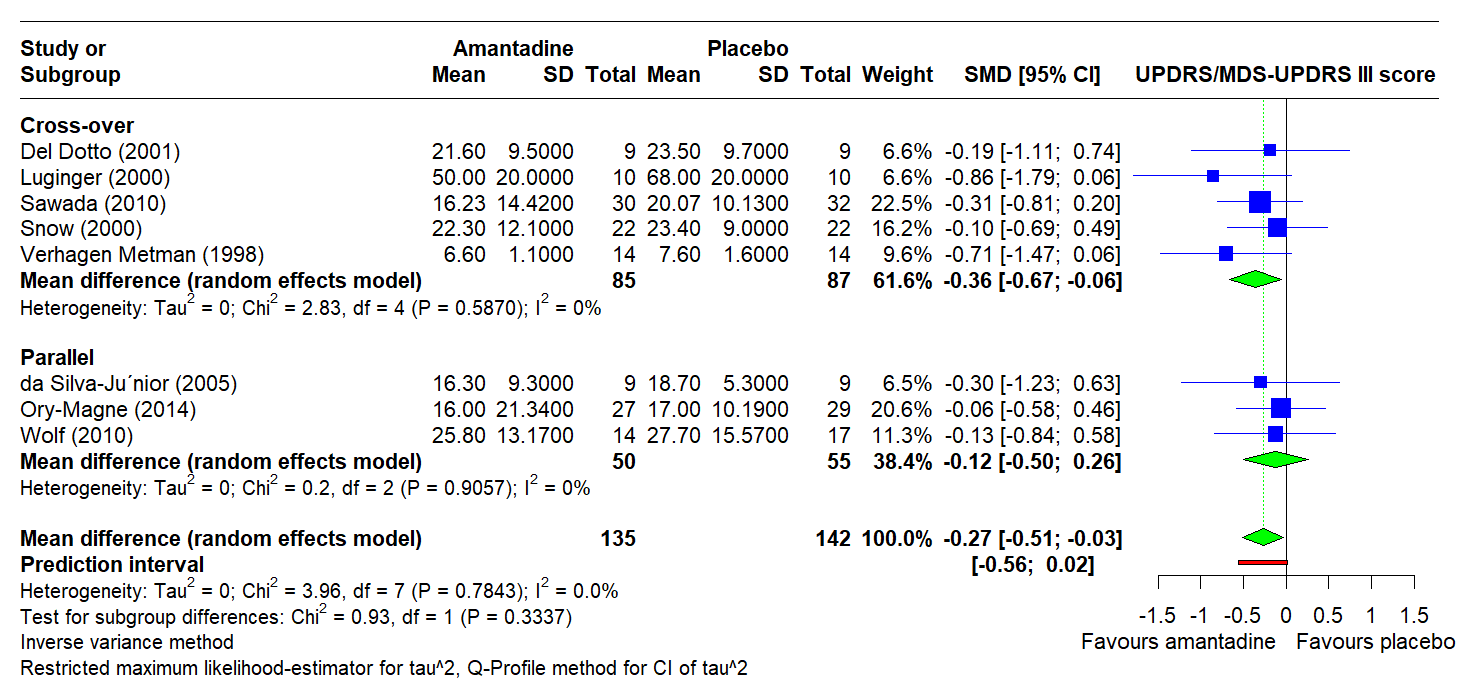


**a)**

**b)**

**c)**

**Fig. S2.** *Forest plots showing the Standardised Mean Differences (SMDs) for the effect of Amantadine compared to placebo on the UPDRS/MDS-UPDRS III score*. ***a)*** *shows the overall effect at up to 13 weeks, and* ***b)*** *and* ***c)*** *show sub-group analyses for the same time point by dosage and trial design respectively. Due to lack of data availability and normality concerns, further analysis was not performed for UPDRS/MDS-UPDRS III.*

*UPDRS/MDS-UPDRS IV: Subgroup Analyses for Dosage and Trial Design*


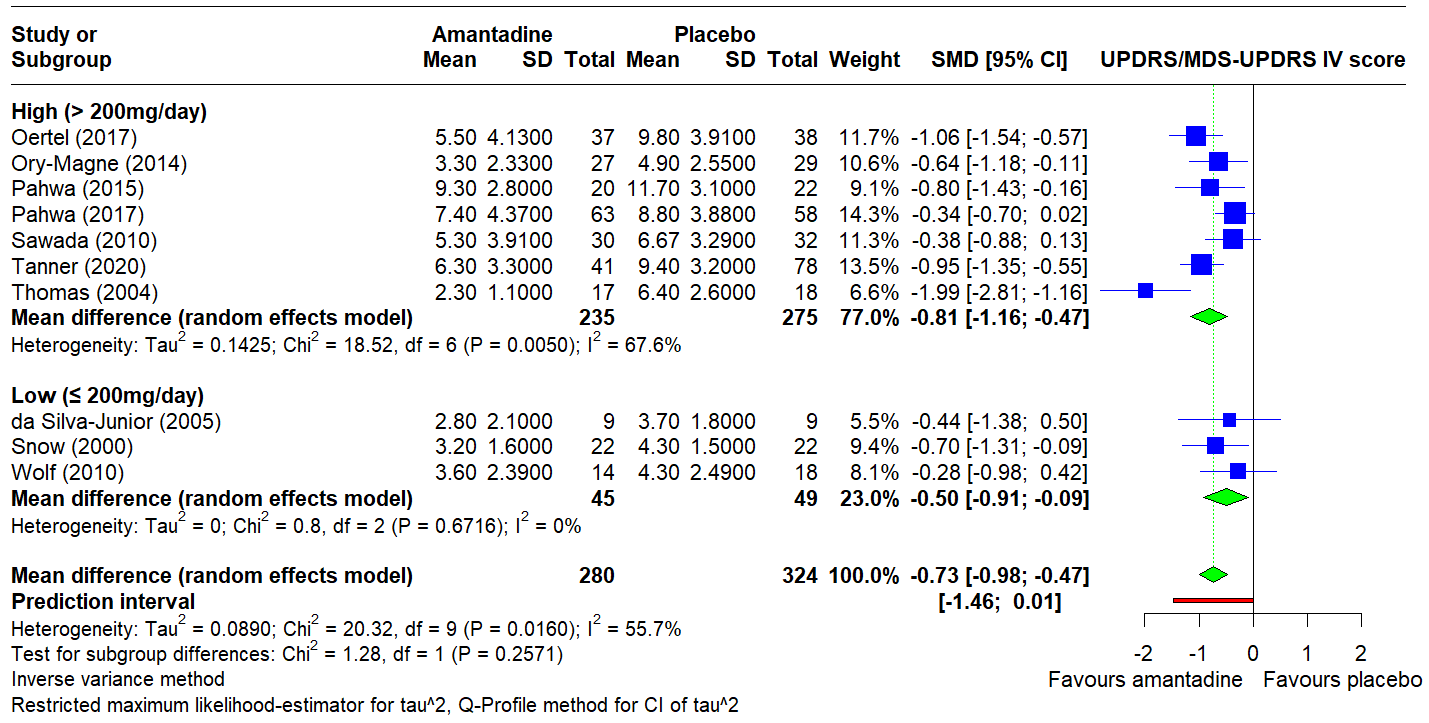

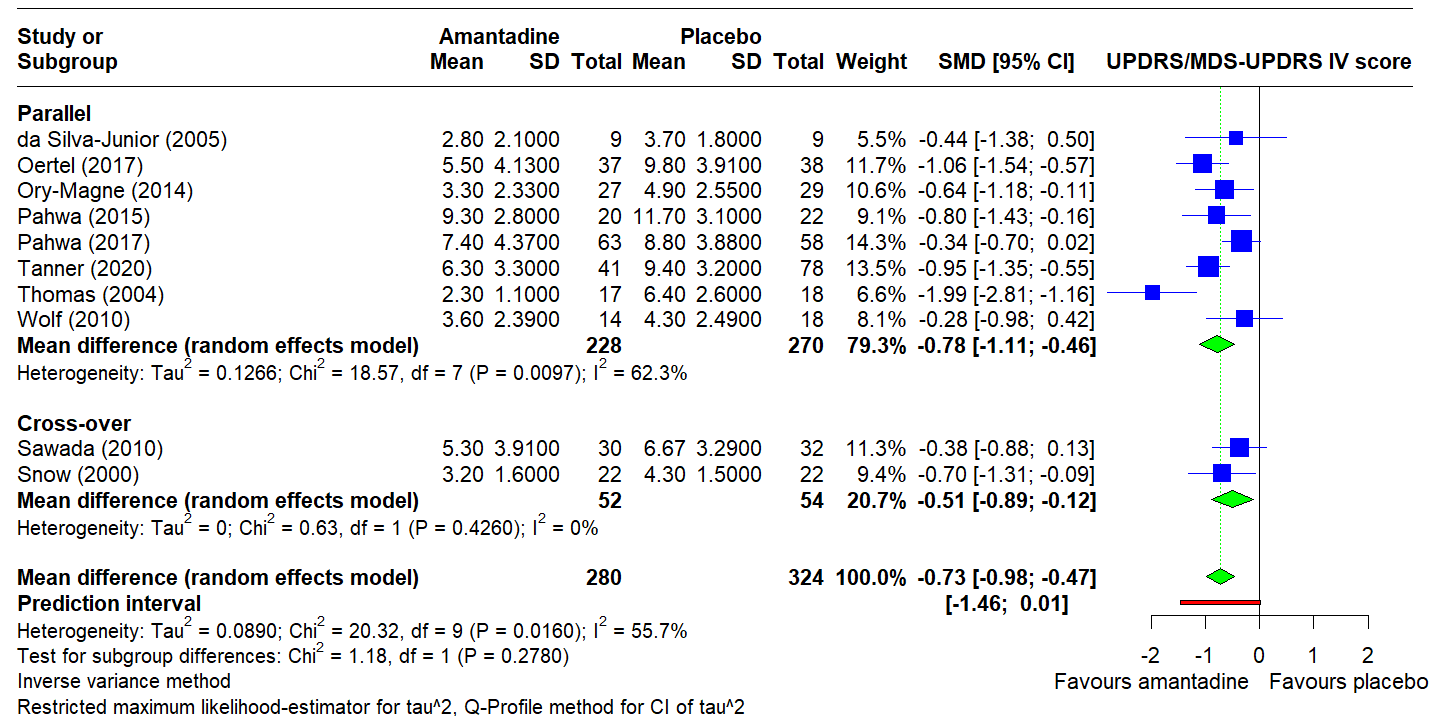


**a)**

**b)**

**Fig. S3.** *Forest plots showing the Standardised Mean Differences (SMDs) for the effect of Amantadine compared to placebo on the UPDRS/MDS-UPDRS IV score at up to 16 weeks of treatment, sub-grouped by* ***a)*** *dosage and* ***b)*** *trial design (cross-over or parallel-group).*

*UPDRS/MDS-UPDRS IV: Subgroup Analysis for Amantadine Formulation*

*
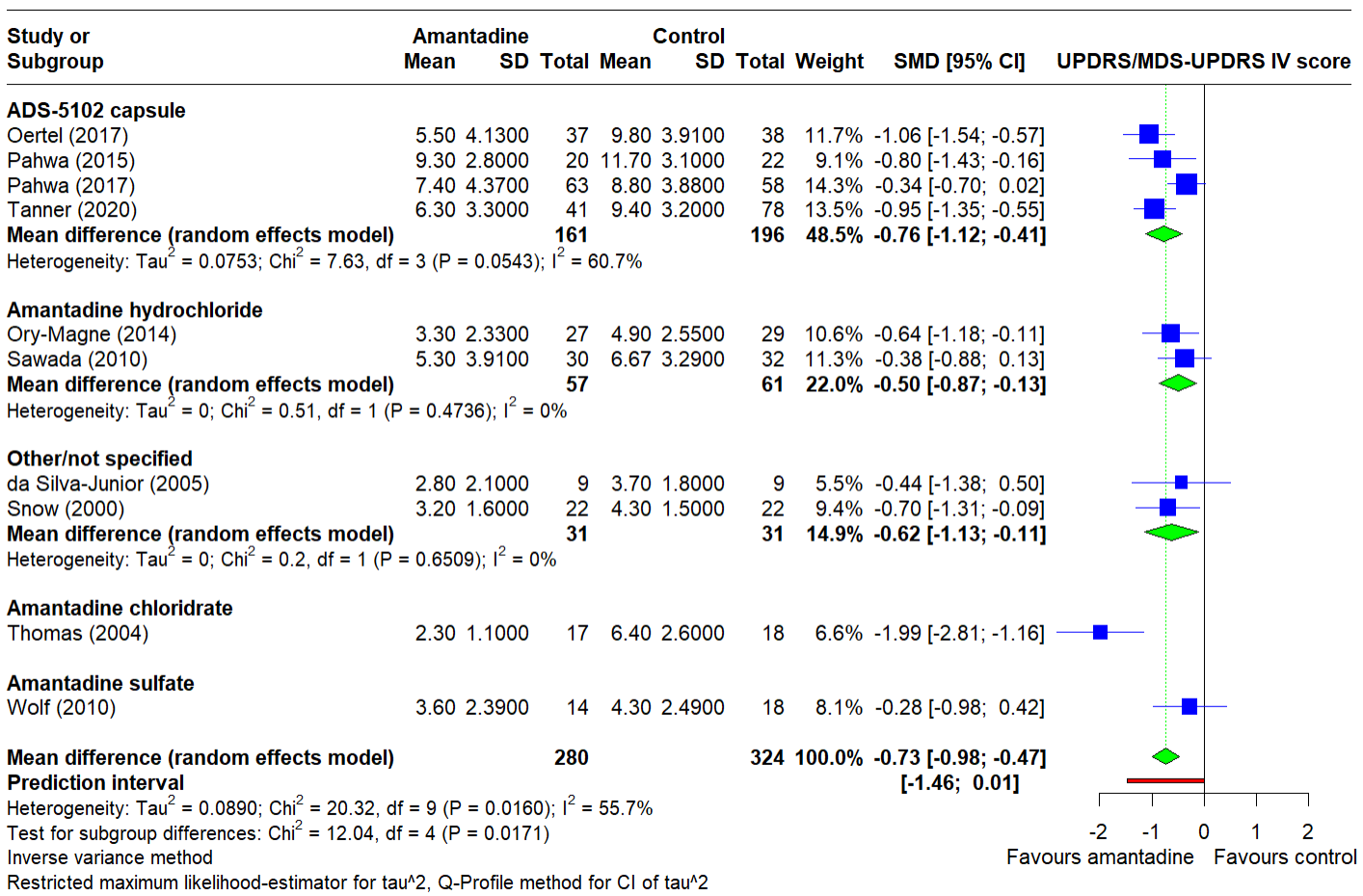
*

**Fig. S4.** *Forest plot showing the Standardised Mean Differences (SMDs) for the effect of Amantadine compared to placebo on the UPDRS/MDS-UPDRS IV score at up to 16 weeks of treatment, sub-grouped by Amantadine formulation (ADS-5102 capsule, Amantadine hydrochloride, Amantadine chloridrate, Amantadine sulfate, or other/unspecified).*

*UPDRS/MDS-UPDRS IV: Sensitivity Analyses*

**a)**

**b)**


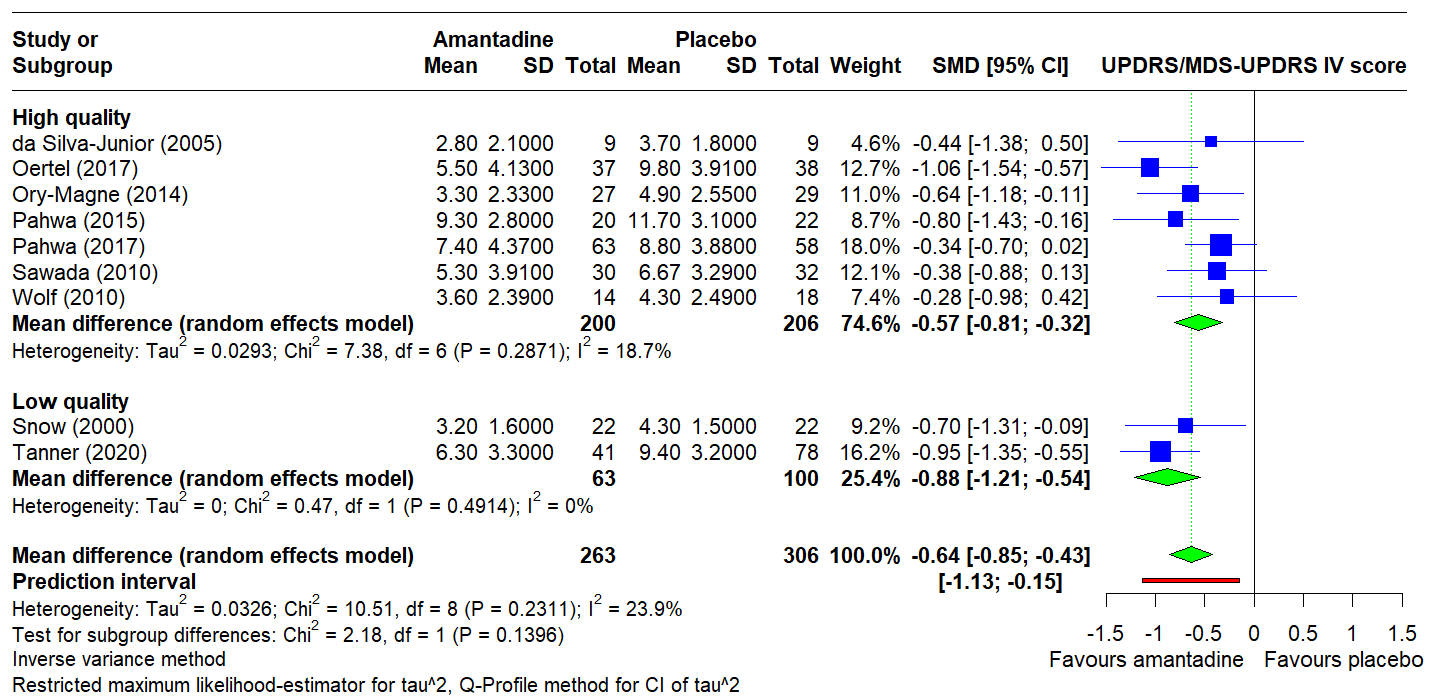

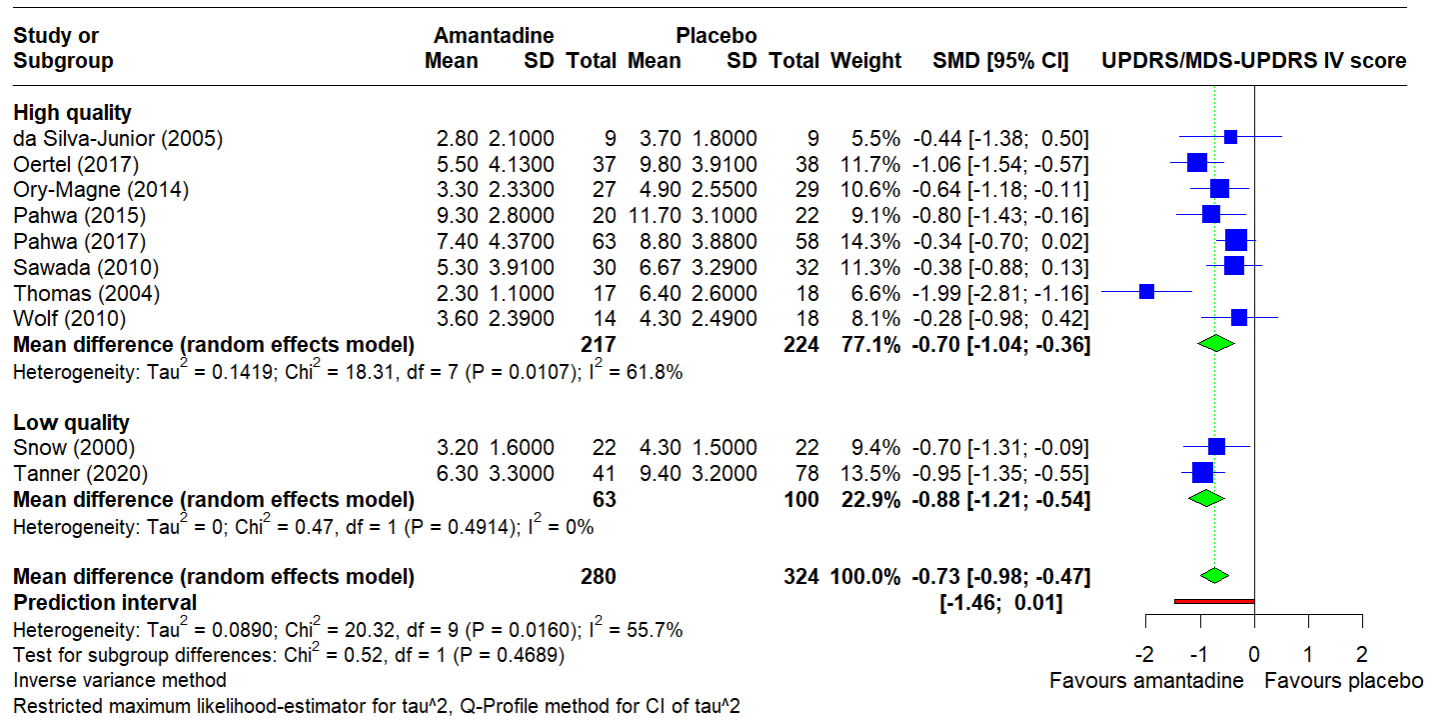


**Fig. S5.** *Forest plot showing the Standardised Mean Differences (SMDs) for the effect of Amantadine compared to placebo on the UPDRS/MDS-UPDRS IV score for up to 16 weeks of treatment, sub-grouped by* *relative study quality (“high” or “low” based on the Cochrane RoB 2 criteria)****.*** *Thomas (2004) has been included in* ***a)*** *but excluded in* ***b)*** *as we believe it may contribute to non-normality in the analysis of UPDRS/MDS-UPDRS IV at 24-38 weeks.*


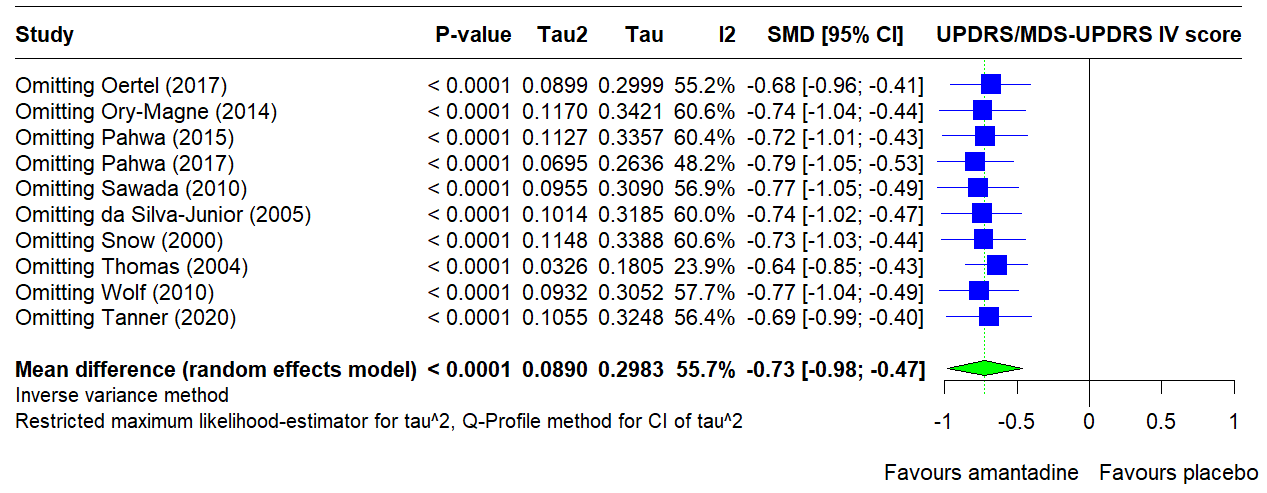


**Fig. S6.** *Forest plot showing the result of “leave-one-out” sensitivity analyses for the effect of Amantadine compared to placebo on the UPDRS/MDS-UPDRS IV score at up to 16 weeks (Standard Mean Differences).*

*UPDRS/MDS-UPDRS IVa*


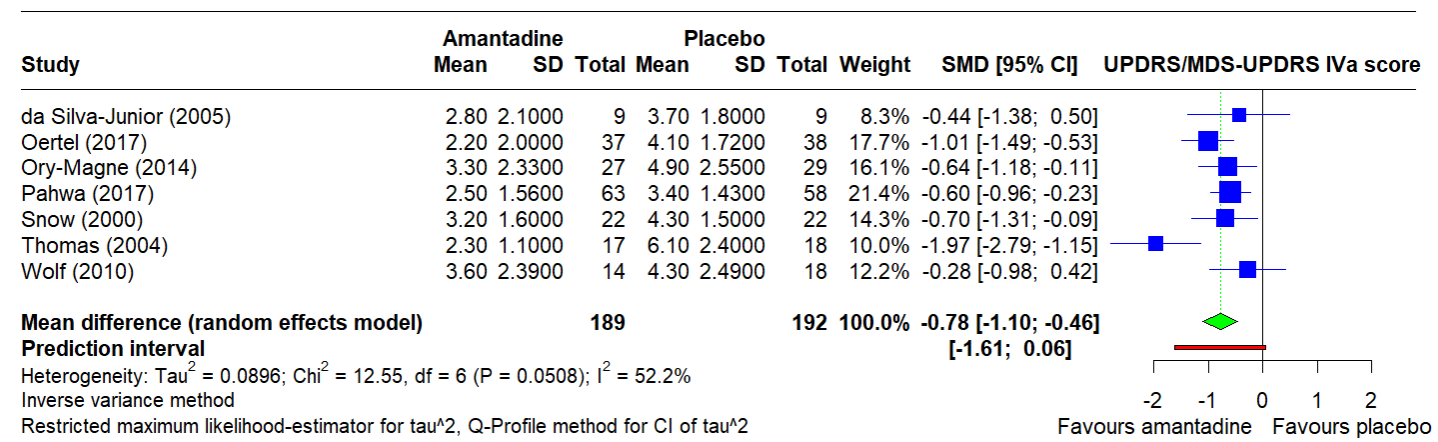

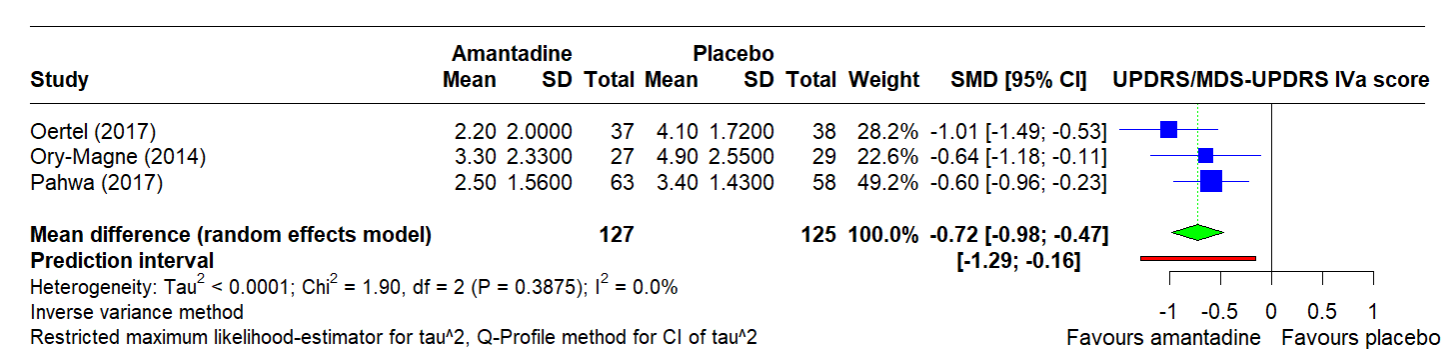


**a)**

**b)**

**Fig. S7.** *Forest plots showing the Standardised Mean Differences (SMDs) for the effect of Amantadine compared to placebo on the UPDRS/MDS-UPDRS IVa score for* ***a)*** *at up to 13 weeks and* ***b)*** *12 to 16 weeks.*

*UDysRS*


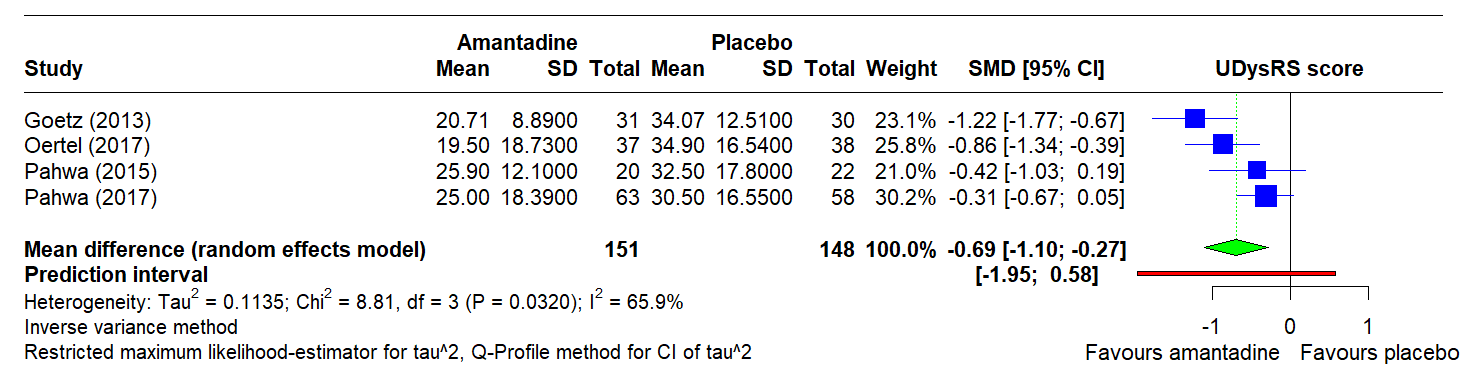

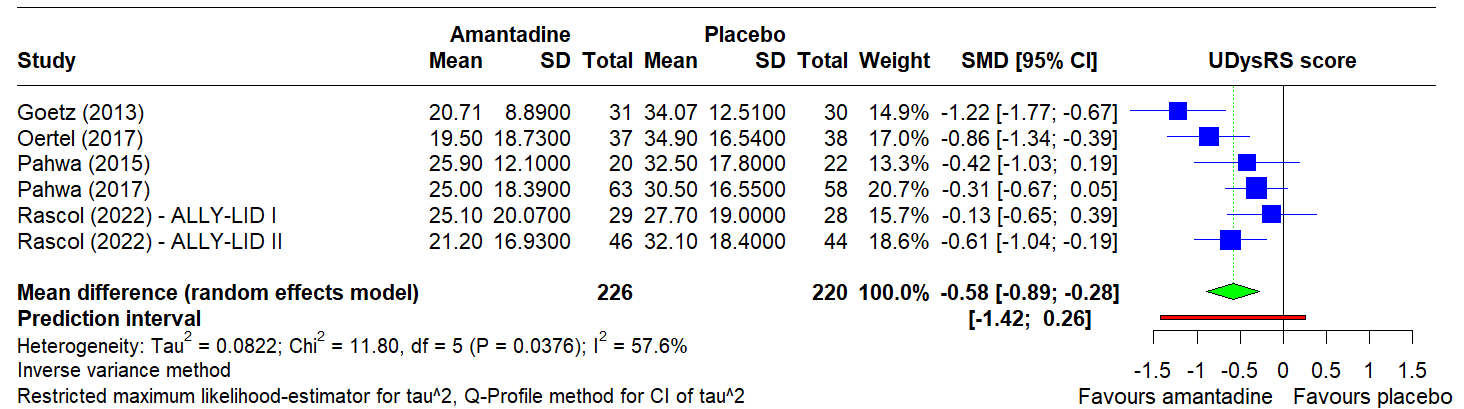

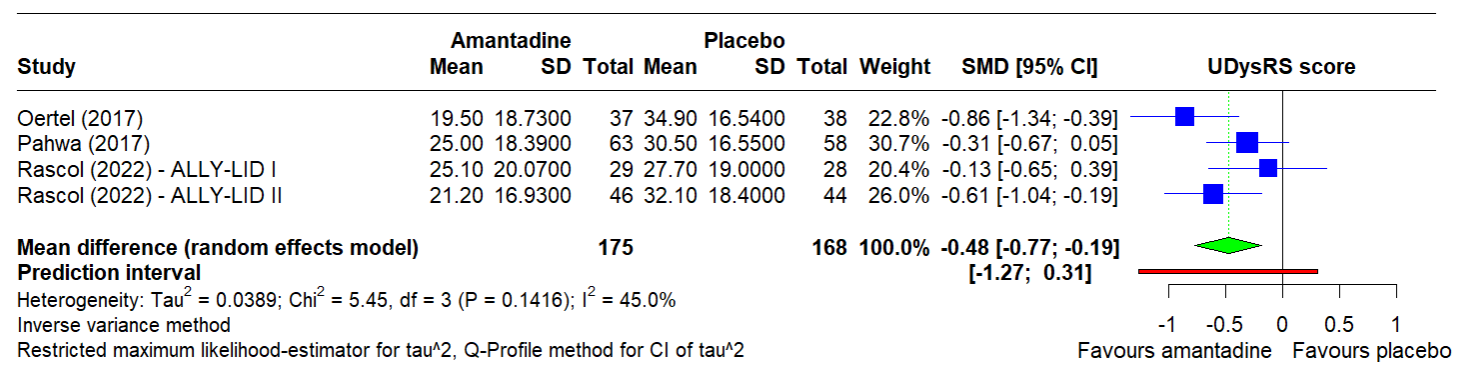


**a)**

**b)**

**c)**

**Fig. S8.** *Forest plots showing the Standardised Mean Differences (SMDs) for the effect of Amantadine compared to placebo on the UDysRS score for* ***a)*** *at up to 13 weeks,* ***b)*** *at up to 16 weeks, and* ***c)*** *12 to 16 weeks.*

*Duration of “OFF” Time*


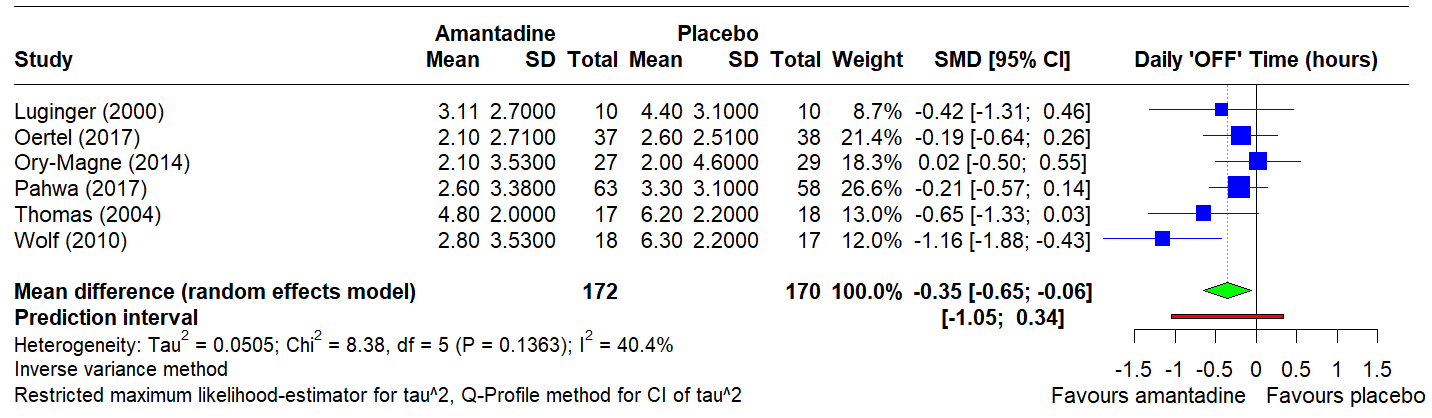

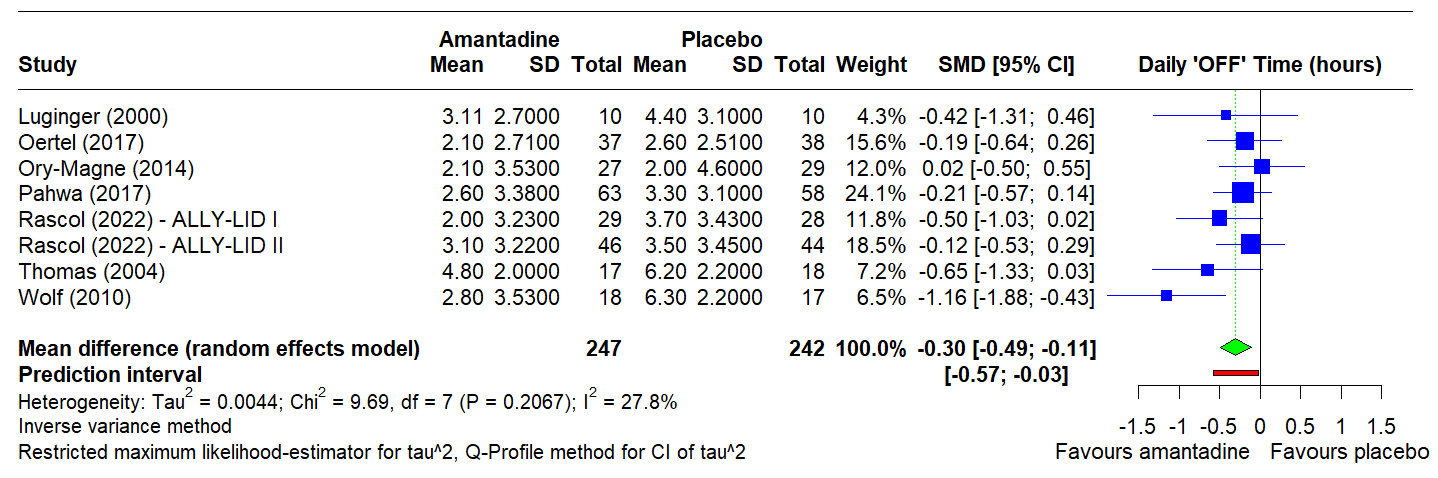

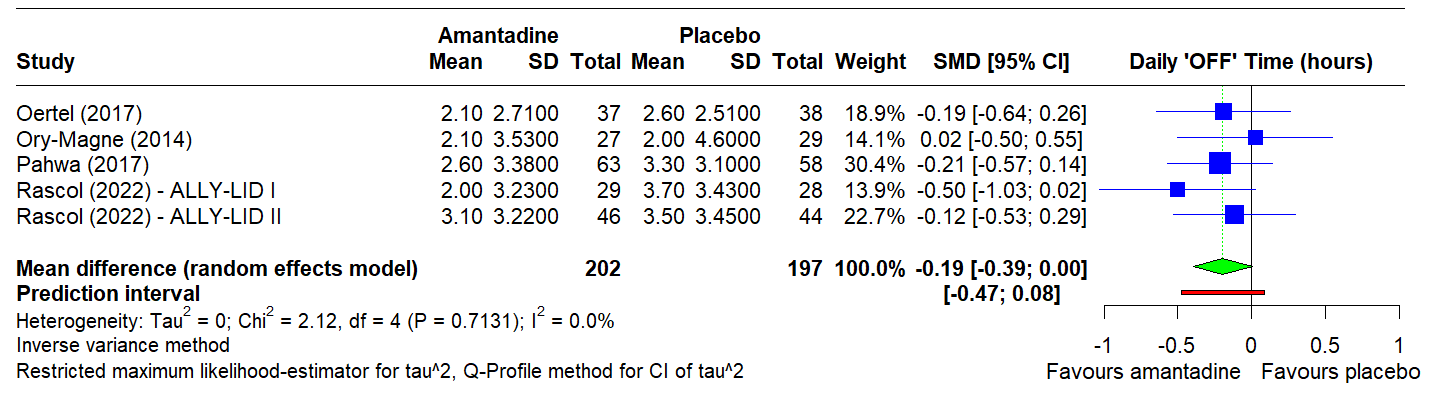


**a)**

**b)**

**c)**

**Fig. S9.** *Forest plots showing the Standardised Mean Differences (SMDs) for the effect of Amantadine compared to placebo on total daily “OFF” time (hours) at* ***a)*** *at up to 13 weeks,* ***b)*** *at up to 16 weeks, and* ***c)*** *12 to 16 weeks.*

**Meta-Analysis Funnel Plot**


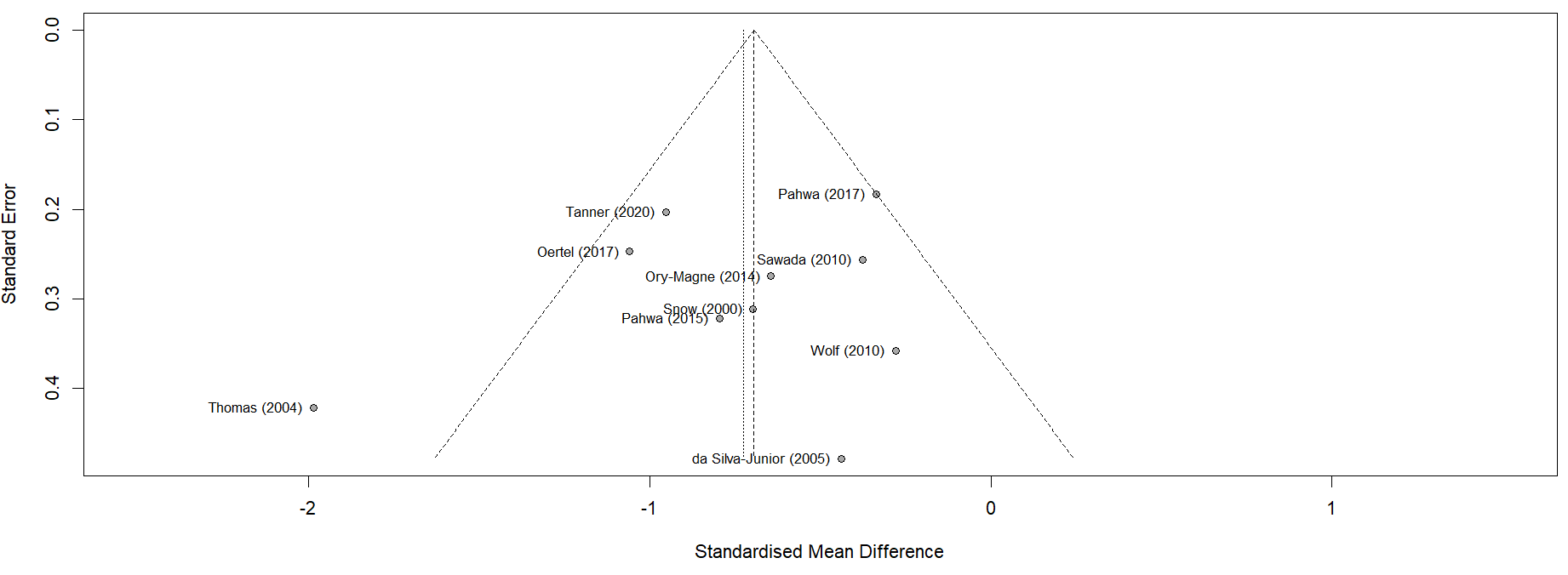


**Fig. S10.** *Funnel/publication bias assessment plot for the effect of Amantadine on UPDRS/MDS-UPDRS IV score at up to 16 weeks. The Cochrane Handbook for Systematic Reviews and its Risk of Bias 2 (RoB 2) tool were used to assess overall study quality and risk of bias. Del Dotto (2001), Wolf (2010), and Goetz (2013) were identified to have a low overall risk of bias. Eight studies – Luginger (2000), Thomas (2004), da Silva-Júnior (2005), Sawada (2010), Ory-Magne (2014), Pahwa (2015), Oertel (2017), Pahwa (2017), and Rascol (2022) – were identified to be of “some concern” due to minor issues in at least one domain, such as missing data or bias in selecting the reported outcomes. We determined a high risk of bias for: Verhagen Metman (1998), due to high risk of missing data; Snow (2000), due to minor bias in three domains (selection bias of reported outcomes, missing data, and randomization); and Tanner (2020), due to a lack of randomization and allocation blinding.*

| **Outcome** | **Time point** | **Effect** | **Significance** | **Clinically meaningful*** | **Meaning to patient** |
| --- | --- | --- | --- | --- | --- |
| **UPDRS/MDS-UPDRS III**  (used to assess motor functions – facial recognition, gait, rigidity, speech) | Up to 13 weeks | -0.27 points | *P* = 0.027 | Yes | After taking Amantadine for 4 months, my motor functions such as facial recognition, gait, rigidity, and speech improve very slightly but not at the level that I or my caregiver would notice. |
| **UPDRS/MDS-UPDRS IV**  (used to assess motor complications – dyskinesias and dystonia) | Up to 13 weeks | -0.69 points | *P* < 0.0001 | Yes | Over the first 4-months of taking Amantadine, my dyskinesias (jerky movements) improve by about 0.70 points. A **1-point** difference at the lower end of the spectrum would be the change between having noticeable symptoms that impact on function or having infrequent symptoms with minimal impact on function. At the higher end, a 1-point difference would be the difference between symptoms significantly impairing function, making some activities impossible, and symptoms that considerably interfere with function but activities are still possible (while difficult).  After taking Amantadine for 6 to 9 months, my dyskinesias (jerky movements) improvement level drops to 0.44 points. |
|  | Up to 16 weeks | -0.73 points | *P* < 0.0001 | Yes |  |
|  | 12 to 16 weeks | -0.73 points | *P* < 0.0001 | Yes |  |
|  | 24 to 38 weeks | -0.44 points | *P* = 0.0372 | Yes |  |
|  | 38 to 101 weeks | -0.36 points | *P* = 0.2149 | No |  |
| **UPDRS/MDS-UPDRS IVa**  (used to assess motor fluctuations or unpredictable changes in motor control, having either having good mobility “ON” or reduced mobility “OFF”)) | Up to 13 weeks | -0.78 points | *P* < 0.0001 | Yes | Over the first 4-months of taking Amantadine, my motor fluctuations improve by about 0.75 points. A **1-point** difference would be the change between predicting my OFF times as **mild** (predictable between 51-75% of the time) vs. **moderate** (predictable between 26-50% of the time). |
|  | 12 to 16 weeks | -0.72 points | *P* < 0.0001 | Yes |  |
| **UDysRS**  (evaluates the severity, frequency and impact on dyskinesias on daily life) | Up to 13 weeks | -0.69 points | *P* = 0.0011 | Yes | Over the first 4-months of taking Amantadine, the impact of the jerky movements on my day-to-day life improves slightly by 0.50-0.70 points. A **1-point** decline would be the change between dyskinesias having a **mild impact on all activities** vs. **a slight impact on a few activities** but you can usually perform all activities and participates in all social interactions during dyskinetic periods. |
|  | Up to 16 weeks | -0.58 points | *P* = 0.0002 | Yes |  |
|  | 12 to 16 weeks | -0.48 points | *P* = 0.0013 | Yes |  |
| **“ON” Time with Troublesome Dyskinesia**  (where you can initiate movement, but you also experience uncontrolled involuntary jerky movements) | Up to 13 weeks | -0.41 hours | *P* = 0.0008 | Yes | Over the first 4-months of taking Amantadine, the amount of time in my moving well ON-state that also has jerky movements will decrease by 25mins per day. |
|  | 12 to 16 weeks | -0.42 hours | *P* < 0.0001 | Yes |  |
| **“ON” Time without Troublesome Dyskinesia**  (where you can initiate movement with no or minimal tremors and jerky movements) | Up to 13 weeks | +0.52 hours | *P* < 0.0001 | Yes | Over the first 4-months of taking Amantadine, I will have about 30mins more per day of being able to move well without having jerky movements. This is good for me to get ready or during my physical exercise routine. |
|  | 12 to 16 weeks | +0.54 hours | *P* < 0.0001 | Yes |  |
| **“OFF” Time**  (where it feels like the medication is losing effect and your motor symptoms are noticeable e.g. it’s difficult to move, increased slowness or increase tremor) | Up to 13 weeks | -0.35 hours | *P* = 0.0181 | Yes | Over the first 3-months of taking Amantadine, the time I spend in my OFF-state decreases by 20mins per day. Between 3 and 4 months, the time I have in my OFF-state increases by 10mins per day compared to in the first 3-months. |
|  | Up to 16 weeks | -0.30 hours | *P* = 0.0016 | Yes |  |
|  | 12 to 16 weeks | -0.19 hours | *P* < 0.0001 | Yes |  |

**Table S1**: *Outcomes along with time-points, effect size, the significance level, and then whether the result is clinically meaningful (i.e. statistically significant as to be useful to a clinician) and/or meaningful to a patient (i.e. a relatively large effect that can be understood by a patient and could improve their quality of life). Rows shaded in blue represent the primary outcomes.*

**Assuming statistical significance level of* P *< 0.05*

**Adverse Events and Side Effects Summary Table**

| **Known PD symptoms** | **Symptoms increased by Levodopa/placebo (reported AEs)** | **Symptoms reduced by Amantadine** | **Symptoms increased by Amantadine (reported AEs)** | **Evidenced in this publication** | **Known side-effects of Amantadine** |
| --- | --- | --- | --- | --- | --- |
| Dyskinesia | Listed in:   1. Sawada et al. (2010) 2. Wolf et al. (2010) 3. Ory-Magne et al. (2014) 4. Rascol et al. (2022) | Amantadine reduced daily “ON” time with dyskinesia by 0.41 hours compared to Levodopa alone | Listed in:   1. Wolf et al. (2010) 2. Ory-Magne et al. (2014) 3. Rascol et al. (2022) | Ref. Figure 3 |  |
| Muscle stiffness/rigidity |  |  | Listed in:   1. Ory-Magne et al. (2014) |  | Yes |
| Imbalance, falls | Listed in:   1. Sawada et al. (2010) 2. Pahwa et al. (2015) 3. Oertel et al. (2017) 4. Pahwa et al. (2017) 5. Rascol et al. (2022) |  | Listed in:   1. Sawada et al. (2010) 2. Wolf et al. (2010) 3. Goetz et al. (2013) 4. Ory-Magne et al. (2014) 5. Pahwa et al. (2015) 6. Oertel et al. (2017) 7. Pahwa et al. (2017) 8. Tanner et al. (2020) 9. Rascol et al. (2022) |  | Yes |
| “ON” |  |  | Amantadine increased daily “ON” time without dyskinesia by 0.52 hours compared to Levodopa alone | Ref. Figure 3 |  |
| “OFF” | Listed in:   1. Wolf et al. (2010) | Amantadine reduced daily “OFF” time by 0.35 hours compared to Levodopa alone |  | Ref. Supp. Figure S8 |  |
| Hallucinations, psychosis | Listed in:   1. Oertel et al. (2017) 2. Pahwa et al. (2017) 3. Rascol et al. (2022) |  | Listed in:   1. Thomas et al. (2004) 2. Sawada et al. (2010) 3. Goetz et al. (2013) 4. Pahwa et al. (2015) 5. Oertel et al. (2017) 6. Pahwa et al. (2017) 7. Tanner et al. (2020) 8. Rascol et al. (2022) |  | Yes (abnormal dreams) |
| Chronic pain | Listed in:   1. Ory-Magne et al. (2014) |  | Listed in:   1. Ory-Magne et al. (2014) |  |  |
| Confusion | Listed in:   1. Pahwa et al. (2015) 2. Rascol et al. (2022) |  | Listed in:   1. Goetz et al. (2013) 2. Pahwa et al. (2015) 3. Rascol et al. (2022) |  | Yes |
| Sleep disturbances, somnolence (drowsiness) | Listed in:   1. Thomas et al. (2004) 2. Ory-Magne et al. (2014) 3. Rascol et al. (2022) |  | Listed in:   1. Goetz et al. (2013) 2. Ory-Magne et al. (2014) 3. Oertel et al. (2017) 4. Tanner et al. (2020) 5. Rascol et al. (2022) |  | Yes |
| Asthenia/fatigue | Listed in:   1. Ory-Magne et al. (2014) 2. Pahwa et al. (2015) 3. Rascol et al. (2022) |  | Listed in:   1. Ory-Magne et al. (2014) 2. Pahwa et al. (2015) 3. Rascol et al. (2022) |  | Yes |
| Nausea/vomiting | Listed in:   1. Goetz et al. (2013) 2. Ory-Magne et al. (2014) 3. Pahwa et al. (2015) 4. Oertel et al. (2017) 5. Rascol et al. (2022) |  | Listed in:   1. Ory-Magne et al. (2014) 2. Pahwa et al. (2015) 3. Oertel et al. (2017) 4. Tanner et al. (2020) 5. Rascol et al. (2022) |  | Yes |
| Decreased appetite |  |  |  |  | Yes |
| Constipation | Listed in:   1. Pahwa et al. (2015) 2. Pahwa et al. (2017) 3. Rascol et al. (2022) |  | Listed in:   1. Sawada et al. (2010) 2. Goetz et al. (2013) 3. Pahwa et al. (2015) 4. Oertel et al. (2017) 5. Pahwa et al. (2017) 6. Tanner et al. (2020) 7. Rascol et al. (2022) |  | Yes |
| Urinary issues (UTI and/or urinary retention) | Listed in:   1. Goetz et al. (2013) 2. Rascol et al. (2022) |  | Listed in:   1. Goetz et al. (2013) 2. Pahwa et al. (2015) 3. Tanner et al. (2020) 4. Rascol et al. (2022) |  | Yes |
| Oedema | Listed in:   1. Rascol et al. (2022) |  | Listed in:   1. Luginger et al. (2000) 2. Pahwa et al. (2015) 3. Pahwa et al. (2017) 4. Tanner et al. (2020) 5. Rascol et al. (2022) |  | Yes |
| Dizziness | Listed in:   1. Luginger et al. (2000) 2. Thomas et al. (2004) 3. Goetz et al. (2013) 4. Pahwa et al. (2015) |  | Listed in:   1. Goetz et al. (2013) 2. Pahwa et al. (2015) 3. Pahwa et al. (2017) 4. Tanner et al. (2020) 5. Rascol et al. (2022) |  | Yes |
| Shortness of breath |  |  |  |  | Yes |
| Blurred vision |  |  | Listed in:   1. Sawada et al. (2010) 2. Goetz et al. (2013) |  | Yes |
| Mood or mental status changes, anxiety, or depression | Listed in:   1. Pahwa et al. (2017) 2. Rascol et al. (2022) |  | Listed in:   1. Pahwa et al. (2015) 2. Pahwa et al. (2017) 3. Rascol et al. (2022) |  | Yes |
| Lack of interest or concern |  |  |  |  | Yes |
| Impulse control disorder |  |  |  |  | Yes |
| Excessive sweating (hyperhidrosis) | Listed in:   1. Goetz et al. (2013) 2. Ory-Magne et al. (2014) |  | Listed in:   1. Ory-Magne et al. (2014) |  | Yes |
| Skin reactions (such as livedo reticularis) and/or rashes | Listed in:   1. Goetz et al. (2013) |  | Listed in:   1. Thomas et al. (2004) 2. Goetz et al. (2013) 3. Pahwa et al. (2015) 4. Pahwa et al. (2017) 5. Tanner et al. (2020) |  | Yes |
| Dry mouth | Listed in:   1. Oertel et al. (2017) |  | Listed in:   1. Goetz et al. (2013) 2. Pahwa et al. (2015) 3. Oertel et al. (2017) 4. Pahwa et al. (2017) 5. Tanner et al. (2020) 6. Rascol et al. (2022) |  | Yes |
| Headache/cephalgia | Listed in:   1. Ory-Magne et al. (2014) 2. Pahwa et al. (2015) 3. Rascol et al. (2022) |  | Listed in:   1. Ory-Magne et al. (2014) 2. Pahwa et al. (2015) 3. Rascol et al. (2022) |  | Yes |
| Orthostatic hypotension | Listed in:   1. Rascol et al. (2022) |  | Listed in:   1. Oertel et al. (2017) 2. Rascol et al. (2022) |  | Yes |

***Table S1****: Adverse events, side effects, and added “adverseness” from Levodopa (placebo) and Amantadine treatment in the analysed studies. For each event, the columns indicate an increase or decrease in their incidence for participants taking either Levodopa only or Amantadine. These are included either as findings from our meta-analysis or a list of the respective articles which reported the AEs as part of their analysis. Columns shaded in blue represent symptoms increased by either Levodopa (placebo) or Amantadine as reported by the included studies.*

**Shapiro-Wilk Normality Testing Results Table**

| **Meta-analysis ref** | **Number of contributing studies** | **Total sample size (N)** | **Shapiro-Wilk W-statistic (3 d.p.)** | **Shapiro-Wilk *P*-value (2 d.p.)** | **Normality satisfied?*** |
| --- | --- | --- | --- | --- | --- |
| UPDRS/MDS-UPDRS III up to 13 weeks (Supplementary Fig S2.a) | 8 | 277 | *Intervention*: 0.83  *Placebo*: 0.73 | *Intervention*: 0.06  *Placebo*: 0.01 | No |
| UPDRS/MDS-UPDRS IV up to 13 weeks (Fig 3.a) | 9 | 485 | *Intervention*: 0.89  *Placebo*: 0.91 | *Intervention*: 0.18  *Placebo*: 0.30 | Yes |
| UPDRS/MDS-UPDRS IV up to 16 weeks (Fig 3.b) | 10 | 604 | *Intervention*: 0.92  *Placebo*: 0.92 | *Intervention*: 0.35  *Placebo*: 0.35 | Yes |
| UPDRS/ MDS-UPDRS IV up to 16 weeks – excluding Thomas (2004) in sensitivity analyses (Fig S4.b) | 9 | 569 | *Intervention:* 0.91  *Placebo*: 0.90 | *Intervention*: 0.35  *Placebo*: 0.25 | Yes |
| UPDRS/MDS-UPDRS IV 12-16 weeks (Fig 3.c) | 4 | 371 | *Intervention*: 0.97  *Placebo*: 0.79 | *Intervention*: 0.81  *Placebo*: 0.08 | Yes |
| UPDRS/MDS-UPDRS IV 24-38 weeks (Fig 3.d) | 3 | 275 | *Intervention*: 0.75  *Placebo*: 0.78 | *Intervention*: 2.20x10^-16^  *Placebo*: 0.06 | No |
| UPDRS/MDS-UPDRS IVa up to 13 weeks (Supplementary Fig S6.a) | 7 | 381 | *Intervention*: 0.94  *Placebo*: 0.91 | *Intervention*: 0.59  *Placebo*: 0.38 | Yes |
| UPDRS/MDS-UPDRS IVa 12-16 weeks (Supplementary Fig S6.b) | 3 | 252 | *Intervention*: 0.94  *Placebo*: >0.99 | *Intervention*: 0.51  *Placebo*: 0.93 | Yes |
| UDysRS up to 13 weeks (Supplementary Fig S7.a) | 4 | 299 | *Intervention*: 0.88  *Placebo*: 0.96 | *Intervention*: 0.34  *Placebo*: 0.77 | Yes |
| UDysRS up to 16 weeks (Supplementary Fig S7.b) | 6 | 446 | *Intervention*: 0.86  *Placebo*: 0.95 | *Intervention*: 0.19  *Placebo*: 0.77 | Yes |
| UDysRS 12-16 weeks (Supplementary Fig S7.c) | 4 | 343 | *Intervention*: 0.85  *Placebo*: >0.99 | *Intervention*: 0.24  *Placebo*: >0.99 | Yes |
| Daily “ON” Time with Troublesome Dyskinesia up to 13 weeks (Fig 4.a) | 4 | 283 | *Intervention*: 0.92  *Placebo*: 0.89 | *Intervention*: 0.51  *Placebo*: 0.41 | Yes |
| Daily “ON” Time with Troublesome Dyskinesia 12-16 weeks (Fig 4.b) | 5 | 399 | *Intervention*: 0.96  *Placebo*: 0.93 | *Intervention*: 0.78  *Placebo*: 0.59 | Yes |
| Daily “ON” Time without Troublesome Dyskinesia up to 13 weeks (Fig 4.c) | 4 | 283 | *Intervention*: 0.99  *Placebo*: 0.97 | *Intervention*: 0.95  *Placebo*: 0.84 | Yes |
| Daily “ON” Time without Troublesome Dyskinesia 12-16 weeks (Fig 4.d) | 3 | 252 | *Intervention*: >0.99  *Placebo*: 0.98 | *Intervention*: 0.94  *Placebo*: 0.75 | Yes |
| Daily “OFF” Time up to 13 weeks (Supplementary Fig S8.a) | 6 | 342 | *Intervention*: 0.82  *Placebo*: 0.90 | *Intervention*: 0.10  *Placebo*: 0.38 | Yes |
| Daily “OFF” Time up to 16 weeks (Supplementary Fig S8.b) | 8 | 489 | *Intervention*: 0.83  *Placebo*: 0.91 | *Intervention*: 0.06  *Placebo*: 0.36 | Yes |
| Daily “OFF” Time 12-16 weeks (Supplementary Fig S8.c) | 5 | 399 | *Intervention*: 0.84  *Placebo*: 0.91 | *Intervention*: 0.15  *Placebo*: 0.50 | Yes |

***Table S2:*** *Results of Shapiro-Wilk normality testing for each meta-analysis included in the current study (a separate test was conducted for the pooled intervention and placebo groups in each analysis to preserve potential differences in distribution). Each meta-analysis is accompanied by the number of studies included, the total sample size, the W-statistic and* P*-value for each test, and whether the analysis satisfied normality assumptions. Note that the UPDRS/MDS-UPDRS IV at 38-101 weeks was excluded as the Shapiro-Wilk test cannot be conducted on data from only two studies.*

**Assuming statistical significance level of* P *< 0.05*
